# Bifunctional anti-PD-L1/TGF-βRII agent SHR-1701 in advanced solid tumors: a dose-escalation, dose-expansion, and clinical-expansion phase 1 trial

**DOI:** 10.1186/s12916-022-02605-9

**Published:** 2022-10-25

**Authors:** Dan Liu, Jun Zhou, Yongsheng Wang, Mingjun Li, Haiping Jiang, Yunpeng Liu, Xianli Yin, Minghua Ge, Xiaojun Xiang, Jieer Ying, Jian Huang, Yan-qiao Zhang, Ying Cheng, Zhigang Huang, Xianglin Yuan, Weiqing Han, Dong Yan, Xinshuai Wang, Pan Liu, Linna Wang, Xiaojing Zhang, Suxia Luo, Tianshu Liu, Lin Shen

**Affiliations:** 1 Key Laboratory of Carcinogenesis and Translational Research (Ministry of Education), Early Drug Development Center, Peking University Cancer Hospital & Institute, Beijing, China; 2 Key Laboratory of Carcinogenesis and Translational Research (Ministry of Education), Department of Gastrointestinal Oncology, Peking University Cancer Hospital & Institute, Fu-Cheng Road 52, Hai-Dian District, Beijing, 100142 China; 3 Oncology, West China Hospital, Sichuan University, Chengdu, China; 4 Oncology Department, The First Affiliated Hospital of Zhengzhou University, Zhengzhou, China; 5 Department of Medical Oncology, First Affiliated Hospital, College of Medicine, Zhejiang University, Hangzhou, China; 6 Medical Oncology, The First Hospital of China Medical University, Shenyang, China; 7 Department of Gastroenterology, Hunan Cancer Hospital, Changsha, China; 8 Head and Neck Surgery, Zhejiang Provincial People’s Hospital, Hangzhou, China; 9 Oncology Department, The First Affiliated Hospital of Nanchang University, Nanchang, China; 10 Hepatobiliary Pancreatic Gastroenterology, Zhejiang Cancer Hospital, Hangzhou, China; 11 Urinary Surgery, Sun Yat-sen Memorial Hospital, Sun Yat-sen University, Guangzhou, China; 12 Ward 2, Department of Gastroenterology, Harbin Medical University Cancer Hospital, Harbin, China; 13 Oncology Department, Jilin Cancer Hospital, Changchun, China; 14 Otolaryngology Head and Neck Surgery Center, Beijing Tongren Hospital, Beijing, China; 15 Oncology Department, Tongji Hospital, Tongji Medical College, Huazhong University of Science and Technology, Wuhan, China; 16 Urology Surgery, Hunan Cancer Hospital (The Affiliated Cancer Hospital of Xiangya School of Medicine, Central South University), Changsha, China; 17 Oncology Department, Beijing Luhe Hospital, Capital Medical University, Beijing, China; 18 Oncology Department, Henan Key Laboratory of Cancer Epigenetics, Cancer Hospital, The First Affiliated Hospital, College of Clinical Medicine, Medical College of Henan University of Science and Technology, Luoyang, China; 19 Clinical Research & Development, Jiangsu Hengrui Pharmaceuticals Co., Ltd, Shanghai, China; 20 Department of Gastroenterology, Henan Tumor Hospital, Dongming Road 127, Jinshui District, Zhengzhou, 450003 China; 21 Medical Oncology, Zhongshan Hospital Fudan University, Fenglin Road 180, Xuhui District, Shanghai, 200032 China

**Keywords:** PD-L1, TGF-β, SHR-1701, Tumor, Immunotherapy

## Abstract

**Background:**

Dual inhibition of PD-1/PD-L1 and TGF-β pathways is a rational therapeutic strategy for malignancies. SHR-1701 is a new bifunctional fusion protein composed of a monoclonal antibody against PD-L1 fused with the extracellular domain of TGF-β receptor II. This first-in-human trial aimed to assess SHR-1701 in pretreated advanced solid tumors and find the population who could benefit from SHR-1701.

**Methods:**

This was a dose-escalation, dose-expansion, and clinical-expansion phase 1 study. Dose escalation was initiated by accelerated titration (1 mg/kg q3w; intravenous infusion) and then switched to a 3+3 scheme (3, 10, 20, and 30 mg/kg q3w and 30 mg/kg q2w), followed by dose expansion at 10, 20, and 30 mg/kg q3w and 30 mg/kg q2w. The primary endpoints of the dose-escalation and dose-expansion parts were the maximum tolerated dose and recommended phase 2 dose. In the clinical-expansion part, selected tumors were enrolled to receive SHR-1701 at the recommended dose, with a primary endpoint of confirmed objective response rate (ORR).

**Results:**

In total, 171 patients were enrolled (dose-escalation: *n*=17; dose-expansion, *n*=33; clinical-expansion, *n*=121). In the dose-escalation part, no dose-limiting toxicity was observed, and the maximum tolerated dose was not reached. SHR-1701 showed a linear dose-exposure relationship and the highest ORR at 30 mg/kg every 3 weeks, without obviously aggravated toxicities across doses in the dose-escalation and dose-expansion parts. Combined, 30 mg/kg every 3 weeks was determined as the recommended phase 2 dose. In the clinical-expansion part, SHR-1701 showed the most favorable efficacy in the gastric cancer cohort, with an ORR of 20.0% (7/35; 95% CI, 8.4–36.9) and a 12-month overall survival rate of 54.5% (95% CI, 29.5–73.9). Grade ≥3 treatment-related adverse events occurred in 37 of 171 patients (22%), mainly including increased gamma-glutamyltransferase (4%), increased aspartate aminotransferase (3%), anemia (3%), hyponatremia (3%), and rash (2%). Generally, patients with PD-L1 CPS ≥1 or pSMAD2 histochemical score ≥235 had numerically higher ORR.

**Conclusions:**

SHR-1701 showed an acceptable safety profile and encouraging antitumor activity in pretreated advanced solid tumors, especially in gastric cancer, establishing the foundation for further exploration.

**Trial registration:**

ClinicalTrials.gov, NCT03710265

**Supplementary Information:**

The online version contains supplementary material available at 10.1186/s12916-022-02605-9.

## Background

Immune checkpoint inhibitors targeting programmed death receptor 1 (PD-1) or its ligand (PD-L1) have been approved for treating multiple advanced or metastatic tumors. However, the objective response rate (ORR) in the all-comer population is less than 20% in most tumor types [1]. Local immunosuppressive factors within the tumor microenvironment could induce the resistance to PD-1/PD-L1 blockade [2]. Combination with chemotherapy, antiangiogenic inhibitors, or other immunotherapies are effective strategies to improve the resistance, but with risks of additive toxicities. Bifunctional antibodies have the potential to resolve these issues.

Transforming growth factor-β (TGF-β)-mediated signaling promotes tumor cell invasiveness, migration, and metastasis [3]. In addition, TGF-β is crucial to create an immune suppressive tumor microenvironment, which inhibits T lymphocyte proliferation, induces naïve T cell’s differentiation into Tregs and Treg expansion, reduces the production of natural killer cells, promotes the differentiation and expansion of myeloid-derived suppressor cells, and consequently enhances immune suppression [4–6]. The independent but complementary immunosuppressive functions between PD-1/PD-L1 and TGF-β pathways make dual inhibition of the two pathways a potent therapeutic strategy. Even in the immune-excluded tumors, blocking TGF-β can enable therapeutic responses to immune checkpoint inhibitors [7].

SHR-1701 is a new bifunctional fusion protein composed of a monoclonal antibody against PD-L1 fused with the extracellular domain of TGF-β receptor II. In vitro and preclinical studies showed that SHR-1701 had a high affinity for PD-L1, TGF-β1, and TGF-β3 and exhibited high PD-L1 target occupancy. We initiated this first-in-human study to assess the safety, tolerability, pharmacokinetics, pharmacodynamics, and preliminary antitumor activity of SHR-1701 in multiple advanced solid tumors.

## Methods

### Study design and participants

This was a multicenter, first-in-human, 3-part, phase 1 trial of SHR-1701 done at 19 hospitals in China. The study was composed of dose-escalation and dose-expansion parts in advanced solid tumors, followed by a clinical-expansion part in selected tumors including biliary tract cancer (BTC), head and neck squamous cell carcinoma (HNSCC), gastric cancer (GC), hepatocellular carcinoma (HCC), pancreatic cancer, renal cell carcinoma (RCC), urothelial carcinoma (UC), and esophageal cancer (ClinicalTrials.gov, NCT03710265; Additional file 1: Fig. S1).

Patients were eligible for dose-escalation and dose-expansion parts if they had pathologically confirmed advanced or metastatic solid tumors that had progressed on or were intolerant to standard therapies or for which no standard therapies were available. Patients enrolled in the clinical-expansion cohort of BTC should (1) have progressed on or are intolerant to at least one line of systemic treatment for advanced or metastatic disease or have progressed on or within 6 months after completion of adjuvant therapy; (2) the last regimen before study entry should be gemcitabine combined with platinum- or fluoropyrimidine-based agents. For patients enrolled in other clinical-expansion cohorts, no more than two lines of prior systemic treatments for advanced or metastatic disease were allowed.

Additional inclusion criteria were age between 18 and 75 years, Eastern Cooperative Oncology Group performance status of 0 or 1, at least one measurable lesion according to Response Evaluation Criteria in Solid Tumors (RECIST; v1.1), life expectancy of at least 12 weeks, and adequate hematological, hepatic, and renal function. Patients in clinical-expansion cohorts should provide fresh tumor tissues or archival samples that were obtained within 12 months before study treatment. Key exclusion criteria included prior exposure to any inhibitor against PD-1, PD-L1, CTLA-4, and/or TGF-β; any anti-cancer treatment within 28 days prior to the first study dose; uncontrolled or symptomatic central nervous system metastases; active or a history of autoimmune disease that was expected to relapse; and immunosuppressive therapy within 7 days prior to the first study dose.

The study was approved by the Ethics Committee of each study center and conducted in accordance with the Good Clinical Practice and Declaration of Helsinki. All patients provided written informed consent. All authors had access to the study data and reviewed and approved the final manuscript.

### Procedures

The dose-escalation part was initiated by an accelerated titration design at 1 mg/kg every 3 weeks, in which only one patient was required if none of the following events occurred during the 21-day tolerability observation: (1) grade ≥2 rash, nausea, vomiting, diarrhea, or fatigue lasting ≥3 days after symptomatic treatment, and any other grade ≥2 non-hematological toxicities; (2) grade ≥3 anemia, grade ≥2 decreased platelet count, grade ≥2 decreased neutrophil count, and any other grade ≥3 hematological toxicities. Otherwise, additional two to five patients were needed for dose at 1 mg/kg every 3 weeks. Standard 3+3 escalation scheme was adopted thereafter, at sequential dose levels of 3, 10, 20, and 30 mg/kg every 3 weeks and 30 mg/kg every 2 weeks. After completion of the dose-escalation part, three or four selected dose regimens would be expanded to collect more data. Subsequently, multiple clinical-expansion cohorts were enrolled to further assess the efficacy of SHR-1701 in selected tumors.

SHR-1701 was given as a 0.5- to 1-h intravenous infusion until disease progression, intolerable toxicity, withdrawal by investigator or patient, or study completion. In the absence of intolerable toxicity or cancer-related clinical deterioration, treatment continuation beyond the initial RECIST v1.1–defined progression was permitted. Treatment interruptions were allowed to manage adverse events. If the toxicity had been reduced to grade ≤1 or baseline level, SHR-1701 could be resumed.

Adverse events were evaluated until 90 days after the last dose and graded according to the National Cancer Institute Common Terminology Criteria for Adverse Events v4.03. Tumor response was assessed by investigators according to RECIST v1.1 and modified RECIST 1.1 for immune-based therapeutics (iRECIST) criteria at screening, every 6 weeks during 24 weeks after first administration, and every 9 weeks thereafter. Complete response (CR) or partial response (PR) should be confirmed at a subsequent assessment after at least 4 weeks.

Serum SHR-1701 concentrations were determined by using a validated enzyme-linked immunosorbent assay with a limit of quantitation of 0.100 μg/mL. Pharmacokinetics parameters were determined by non-compartmental analysis.

The PD-L1 target occupancy in peripheral blood mononuclear cells was assessed by flow cytometry. CD3^+^ T lymphocytes were identified using Alexa Fluor 488 mouse anti-human CD3 (BD Biosciences, San Jose, CA, USA) and according to their forward scatter and side scatter features. AF647-SHR-1316 was used to detect the PD-L1 targets not bound by SHR-1701. The cell population was gated on the CD3^+^ T cells and target occupancy was calculated based on the pre-dose samples for each patient. The free TGF-β1 level in plasma was determined by electrochemiluminescence assay (Meso Scale Discovery, MD, USA).

PD-L1 tumor expression was determined by immunohistochemistry carried out at a central laboratory (E1L3N clone, Cell Signaling Technology) and calculated as combined positive score (CPS, defined as the number of PD-L1 staining cells [tumor cells, lymphocytes, and macrophages] out of the total number of tumor cells, multiplied by 100).

Phosphorylation of SMAD2 was centrally detected by immunohistochemistry (138D4 clone, Cell Signaling Technology) and presented as histochemical score (H-score, defined and calculated as the product of the intensity score and proportion) in tumor and immune cells.

### Outcomes

The primary endpoints of dose-escalation and dose-expansion parts were the maximum tolerated dose and recommended phase 2 dose. Dose-limiting toxicities were defined as a treatment-related adverse event that occurred during the first treatment cycle (21 days for every 3 weeks schedule and 28 days for every 2 weeks schedule) and met any of the following criteria: (1) grade ≥3 non-hematological toxicities, excluding grade ≥3 nausea, vomiting, diarrhea, or fatigue that recovered to grade ≤2 within 3 days after symptomatic treatment, transient grade 3 infusion reaction or fever (<6 h), and grade 3 increased alanine aminotransferase, increased aspartate aminotransferase, or skin toxicities that recovered to grade ≤2 within 7 days after appropriate treatment; (2) grade 3 decreased platelet count lasting for ≥7 days or with bleeding symptom, grade 3 anemia that could not recover to ≥9 g/dL within 14 days without blood transfusion or use of erythroid growth factor, grade 3 neutropenic infection or febrile neutropenia (≥38.5°C), grade 4 decreased neutrophil count lasting for ≥4 days, and any other grade ≥4 hematological toxicities; (3) other unexpected, durable, and intolerable grade ≥2 toxicities requiring discontinuation of SHR-1701 as judged by the Safety Monitoring Committee. Maximum tolerated dose was defined as the maximum dose level in which ≤1 of 6 patients experienced a dose-limiting toxicity. The recommended dose was determined by the Safety Monitoring Committee based on all results during the dose-escalation and dose-expansion parts. Second endpoints were the pharmacokinetic profile, pharmacodynamic activity, and preliminary antitumor activity.

The primary endpoint of the clinical-expansion part was confirmed ORR assessed by RECIST v1.1, defined as the percentage of patients whose best overall response was confirmed CR or PR. Second endpoints included disease control rate (DCR), clinical benefit rate (CBR, defined as CR, PR, or stable disease lasting at least 24 weeks), duration of response (DoR), and progression-free survival (PFS) per RECIST v1.1, as well as overall survival (OS).

Exploratory endpoints included efficacy outcomes assessed according to iRECIST and correlations of baseline PD-L1 expression and pSMAD2 activity with tumor response to SHR-1701.

### Statistical analysis

The total number of patients required for dose escalation depended on the toxicities observed, with 3 to 6 patients per dose level except the initial dose. For the dose levels expanded, a total of 10 to 12 patients per dose level were required. For the clinical-expansion cohorts of selected tumors, 20 to 30 patients per cohort were planned.

Efficacy and safety were analyzed in all patients who received at least one dose of study treatment. The population for pharmacokinetic or pharmacodynamic analysis included all patients who received study treatment and had at least one corresponding post-treatment variable.

ORR, DCR, and CBR were reported with the corresponding 95% CI calculated via the Clopper–Pearson method. The Kaplan–Meier method was used to estimate the median DoR, PFS, and OS, and the 95% CIs were estimated by the Brookmeyer–Crowley method. The areas under the curve (AUCs) were generated by plotting receiver operating characteristic curves that illustrated sensitivity and 1-specificity for pSMAD2 level. Fisher’s exact test was used to assess the independence of ORR and pSMAD2 level. Statistical analyses were done using SAS v9.4 and pharmacokinetic analyses were done using Phoenix WinNonlin v8.1.

## Results

### Patients

Between November 13, 2018, and April 30, 2021, a total of 171 patients were enrolled and administered with SHR-1701. The majority of patients had an Eastern Cooperative Oncology Group performance status of 1 (*n*=136, 80%) and received prior systemic therapies (*n*=168, 98%; Table 1). As of the cutoff date on July 30, 2021, 39 (23%) patients remained on study treatment, and 132 (77%) discontinued treatment, mainly due to radiographical progression. Thirty (18%) patients continued SHR-1701 treatment beyond the initial RECIST v1.1–defined progression.

**Table 1:** Demographics and clinical characteristics

	Dose-escalation and expansion part (***N***=50)	Clinical-expansion part	All patients
		(***N***=121)	(***N***=171)
**Age, years**	55 (48–62)	57 (50–65)	56 (49–64)
**Sex**			
Male	30 (60%)	98 (81%)	128 (75%)
Female	20 (40%)	23 (19%)	43 (25%)
**ECOG performance status**			
0	12 (24%)	23 (19%)	35 (20%)
1	38 (76%)	98 (81%)	136 (80%)
**No. of organs of metastases**			
0	1 (2%)	6 (5%)	7 (4%)
1	13 (26%)	38 (31%)	51 (30%)
2	13 (26%)	35 (29%)	48 (28%)
3	10 (20%)	21 (17%)	31 (18%)
4 or more	13 (26%)	21 (17%)	34 (20%)
**Lines of prior anti-cancer therapies**			
0	0	3 (3%)	3 (2%)
1	14 (28%)	78 (64%)	92 (76%)
2	21 (42%)	40 (33%)	61 (50%)
3 or more	15 (30%)	0	15 (9%)
**Prior therapy**			
Chemotherapy	46 (92%)	95 (79%)	141 (82%)
Targeted therapy	25 (50%)	42 (35%)	67 (39%)
Immunotherapy	5 (10%)	1 (<1%)	6 (4%)
Others	5 (10%)	5 (4%)	10 (6%)

*ECOG* Eastern Cooperative Oncology Group. Data are median (IQR) or *n* (%)

### Determination of recommended phase 2 dose

**Fig. 1:**
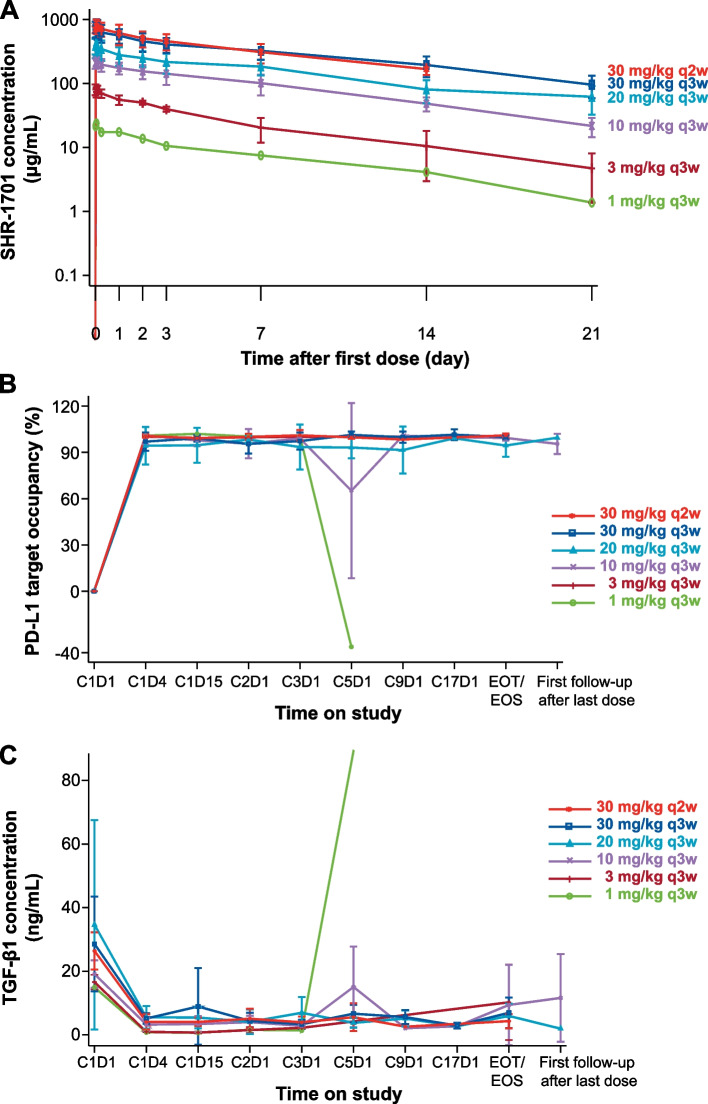
Pharmacokinetic profile and pharmacodynamic activity. **A** Semi-logarithm mean serum concentration–time profiles of SHR-1701. Error bars represent standard deviation. **B** PD-1 target occupancy following SHR-1701 treatment. There was a sharp decrease in the patient in the 1 mg/kg every 3 weeks group on C5D1 before administration, which might be caused by delayed treatment (interval between C4D1 and C5D1, 27 days). The patient withdrew from the study after C5D1. In the 10 mg/kg every 3 weeks group, PD-L1 target occupancy of 1 patient decreased to 25% on C5D1, but reached saturated level before administration on C9D1. All the remaining patients had a sustained and saturated PD-L1 target occupancy throughout the study. **C** TGF-β1 concentrations following SHR-1701 treatment. The free TGF-β1 level in the patient in the 1 mg/kg every 3 weeks group sharply increased on C5D1 before administration. In addition to treatment delay, low dose level might be the reason. PD-L1, programmed death ligand 1; TGF-β1, transforming growth factor-β 1; EOT, end of treatment; EOS, end of study

In the dose-escalation part, no dose-limiting toxicity was observed in the 17 patients, and the maximum tolerated dose was not reached. Subsequently, 10, 20, and 30 mg/kg every 3 weeks and 30 mg/kg every 2 weeks doses were expanded, with another 33 patients enrolled. A linear dose-exposure relationship with SHR-1701 dosing from 1 to 30 mg/kg was observed (Fig. 1A). Pharmacokinetic parameters of SHR-1701 following a single infusion are listed in Additional file 1: Table S1. The concentration of SHR-1701 peaked at 1.68 to 2.98 h after infusion. The geomean half-life ranged from 4.6 to 8.1 days. Parameters reflecting exposure (including C_max_, AUC_last_, and AUC_inf_) increased and clearance decreased slowly over the dose ranges examined. PD-L1 target occupancy rate at the surface of peripheral blood CD3-positive T cells exceeded 90% at 72 h after the first dose (Fig. 1B). At 72 h, the free TGF-β1 levels in peripheral blood sharply decreased. Nearly complete TGF-β1 trapping was detected in all dose groups (Fig. 1C). SHR-1701 at 30 mg/kg every 3 weeks exerted the best antitumor activity, without obviously aggravated toxicities compared with other dose levels (Additional file 1: Fig. S2 and Table S2 showing the data at cutoff date). Combined, 30 mg/kg every 3 weeks was determined as the recommended phase 2 dose.

### Efficacy in select tumors

In the clinical-expansion part, 121 patients were enrolled in eight cohorts and received SHR-1701 at the recommended dose, including 35 with GC, 21 with HCC, 13 with BTC, 12 with UC, 10 with HNSCC, 10 with RCC, 10 with pancreatic cancer, and 10 with esophageal cancer (Additional file 1: Table S3 showing patient characteristics by tumor type).

**Fig. 2:**
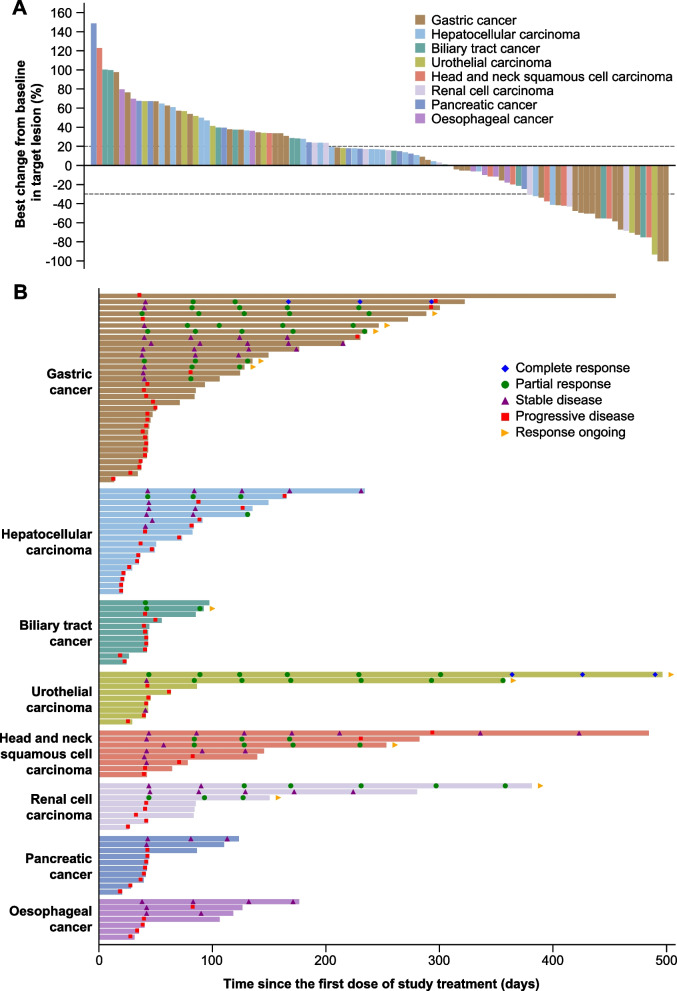
Tumor responses in select tumors at the recommended dose (30 mg/kg every 3 weeks). **A** Best percentage change from baseline in target lesion size. **B** Tumor responses per RECIST v1.1 over time

In total, 15 of the 121 patients achieved confirmed objective responses according to RECIST v1.1 (Table 2), including two CRs (one patient with GC and one with UC) and 13 PRs (six patients with GC, two with HNSCC, two with RCC, one with UC, one with BTC, and one with HCC). Tumor shrinkage in target lesions was observed in 38 of 102 (37%) evaluable patients (Fig. 2A), and a durable response was clearly observed in patients who had a reduction of 30% or more in the target lesion (Additional file 1: Fig. S3). Moreover, as assessed by iRECIST, another two GC patients (2%) achieved objective response from continued SHR-1701 treatment after immune unconfirmed progressive disease (iUPD).

**Table 2:** Objective response determined by RECIST v1.1

	Gastric cancer (***n***=35)	Hepatocellular carcinoma (***n***=21)	Biliary tract cancer (***n***=13)	Urothelial carcinoma (***n***=12)	Head and neck squamous cell carcinoma (***n***=10)	Renal cell carcinoma (***n***=10)	Pancreatic cancer (***n***=10)	Esophageal cancer (***n***=10)
**Best overall response**								
Complete response	1 (3%)	0	0	1 (8 %)	0	0	0	0
Partial response	6 (17 %)	1 (5%)	1 (8%)	1 (8%)	2 (20%)	2 (20%)	0	0
Stable disease	6 (17%)	6 (29%)	1 (8%)	1 (8%)	4 (40%)	1 (10%)	2 (20%)	3 (30%)
Progressive disease	19 (54%)	11 (52%)	9 (69%)	6 (50%)	2 (20%)	5 (50%)	8 (80%)	4 (40%)
Not evaluable	3 (9%)	3 (14%)	2 (15%)	3 (25%)	2 (20%)	2 (20%)	0	3 (30%)
**Objective response rate**	20.0% (8.4–36.9)	4.8% (0.1–23.8)	7.7% (0.2–36.0)	16.7% (2.1–48.4)	20.0% (2.5–55.6)	20.0% (2.5–55.6)	0% (0–30.9)	0% (0–30.9)
**Disease control rate**	37.1% (21.5–55.1)	33.3% (14.6–57.0)	15.4% (1.9–45.5)	25.0% (5.5–57.2)	60.0% (26.2–87.8)	30.0% (6.7–65.3)	20.0% (2.5–55.6)	30.0% (6.7–65.3)
**Clinical benefit rate**	28.6% (14.6–46.3)	9.5% (1.2–30.4)	7.7% (0.2–36.0)	16.7% (2.1–48.4)	30.0% (6.7–65.3)	30.0% (6.7–65.3)	0% (0–30.9)	10.0% (0.3–44.5)

Data are *n* (%) or % (95% CI)

The most favorable efficacy was shown in the GC cohort, with an ORR of 20.0% (95% CI, 8.4–36.9) per RECIST v1.1 and 25.7% (95% CI, 12.5–43.3) per iRECIST. Responses were still ongoing in five of seven responders per RECIST v1.1 (Fig. 2B) and seven of nine responders per iRECIST, and the estimated median DoR was 7.0 months (95% CI, 7.0–not reached) per RECIST v1.1 and 7.1 months (95% CI, 7.0–not reached) per iRECIST, respectively. Median PFS was 1.4 months (95% CI, 1.4–7.5) when determined by RECIST v1.1 and 2.5 months (95% CI, 1.4–9.6) when determined by iRECIST. The 12-month OS rate was 54.5% (95% CI, 29.5–73.9).

Encouraging antitumor activity was also observed in HNSCC, RCC, and UC cohorts, with an ORR of 20.0%, 20.0%, and 16.7% (Table 2), respectively, and the median DoR in these cohorts had not been reached yet.

### Safety

Treatment-related adverse events (TRAEs) of any grade occurred in 120 (70%) of 171 patients (Table 3). Grade 3 or 4 TRAEs were reported in 34 (20%) patients, with increased gamma-glutamyltransferase (six patients, 4%), increased aspartate aminotransferase (five, 3%), anemia (five, 3%), hyponatremia (five, 3%), and rash (four, 2%) occurring in more than two patients. The majority of these events were improved or resolved at data cutoff. Three (2%) grade 5 events were considered to be treatment related by the investigators, including one (<1%) caused by pneumonia and two (1%) unknown deaths. TRAEs led to treatment interruption in 41 (24%) patients. Five (3%) patients permanently discontinued study treatment due to TRAEs including increased lipase, hypersensitivity, lichen planus, type 1 diabetes mellitus, and hyponatremia (one patient, <1% for each). Serious TRAEs occurred in 25 (15%) patients (Additional file 1: Table S4).

**Table 3:** Treatment-related adverse events

	All patients (***N***=171)				
	Any grade	Grade 1	Grade 2	Grade 3	Grade 4
**Any**	120 (70%)	34 (20%)	49 (29%)	27 (16%)	7 (4%)
Aspartate aminotransferase increased	40 (23%)	30 (18%)	5 (3%)	4 (2%)	1 (<1%)
Alanine aminotransferase increased	29 (17%)	22 (13%)	5 (3%)	1 (<1%)	1 (<1%)
Anemia	26 (15%)	9 (5%)	12 (7%)	5 (3%)	0
Hypothyroidism	19 (11%)	9 (5%)	10 (6%)	0	0
Rash	18 (11%)	10 (6%)	4 (2%)	4 (2%)	0
Blood bilirubin increased	18 (11%)	13 (8%)	4 (2%)	1 (<1%)	0
Protein urine present	15 (9%)	6 (4%)	9 (5%)	0	0
Bilirubin conjugated increased	14 (8%)	9 (5%)	3 (2%)	2 (1%)	0
Asthenia	13 (8%)	8 (5%)	4 (2%)	1 (<1%)	0
Gamma-glutamyltransferase increased	12 (7%)	3 (2%)	3 (2%)	5 (3%)	1 (<1%)
Decreased appetite	12 (7%)	9 (5%)	3 (2%)	0	0
Pyrexia	11 (6%)	7 (4%)	4 (2%)	0	0
Pruritus	10 (6%)	7 (4%)	2 (1%)	1 (<1%)	0
Hyponatremia	9 (5%)	4 (2%)	0	4 (2%)	1 (<1%)
Blood alkaline phosphatase increased	9 (5%)	5 (3%)	3 (2%)	1 (<1%)	0
Platelet count decreased	9 (5%)	5 (3%)	4 (2%)	0	0
Gingival bleeding	9 (5%)	5 (3%)	4 (2%)	0	0
Proteinuria	9 (5%)	7 (4%)	2 (1%)	0	0

Data are present as *n* (%). Treatment-related adverse events that occurred in at least 5% of all treated patients are listed. Three (2%) grade 5 events were considered to be treatment related by the investigators, including one (<1%) caused by pneumonia and two (1%) unknown deaths

Study treatment was temporarily stopped due to grade ≥3 TRAEs in 19 (11%) patients. Among them, seven patients were rechallenged with SHR-1701, and only one TRAE recurred in one patient (hyponatremia, grade 4) and eventually resulted in permanent discontinuation of SHR-1701.

Any grade immune-related adverse events assessed by the investigator occurred in 62 (36%) patients, and grade 3 or worse ones occurred in 16 (9%) patients. The most common immune-related adverse events with an incidence of more than 5% were hypothyroidism and rash (17 patients, 10% for each).

### Biomarker analyses

Tumor biospecimens of 112 patients were available for PD-L1 expression assessment (Additional file 1: Table S5). Based on RECIST v1.1, patients with high PD-L1 expression regardless of tumor type showed higher ORR (CPS ≥1 vs <1: 20.8% vs 7.5%; ≥5 vs <5: 34.1% vs 5.6%; ≥10 vs <10: 40.6% vs 6.3%). Similarly, in the GC cohort, ORR per RECIST v1.1 was 21.7% vs 12.5% in patients with PD-L1 CPS ≥1 vs <1; 45.5% vs 5.0% in those with PD-L1 CPS ≥5 vs <5; and 55.6% vs 4.5% in those with PD-L1 CPS ≥10 vs <10.

pSMAD2 levels were available in 43 patients (Additional file 1: Table S6). No obvious associations were observed between pSMAD2 level in immune cells and ORR (AUC=0.48 per RECIST v1.1). In tumor cells, pSMAD2 H-score ≥235 was associated with higher ORR (AUC=0.73; 36.4% vs 6.3%; Additional file 1: Fig. S4).

## Discussion

The present study reported the clinical outcomes of SHR-1701 in pretreated patients with advanced solid tumors, aiming to assess its safety and tolerability and identify the population who could benefit from SHR-1701.

SHR-1701 showed a manageable safety profile with 22% of patients having grade 3 or worse TRAEs, which were similar to bintrafusp alfa (another bifunctional conjugate targeting TGF-β and PD-L1 under investigation) [8]. Squamous cell carcinoma (SCC) of skin and keratoacanthoma were potentially TGF-β–mediated cutaneous events [9–11] and occurred in 4% and 8% of patients treated with bintrafusp alfa, with 2% and <1% being grade 3 or worse in severity [12]. In our study, SHR-1701 treatment did not result in the occurrence of skin SCC or keratoacanthoma. Considering that skin color and exposure to UV radiation were reported to be significant risk factors for keratoacanthoma [13, 14] or skin SCC [15], the differences in the enrolled population and their sunbathing habits were more likely to be the main explanations for the two skin toxicities, in addition to possible distinct actions of the two drugs. Besides, some patients suffered bleeding events following bintrafusp alfa, with the most common being epistaxis (12%), hemoptysis (7%), and gingival bleeding (5%) [12]. In this study of SHR-1701, the bleeding event that occurred in at least 5% of patients was gingival bleeding only, and most bleeding events were grade 1 or 2 in severity. Only one (<1%) patient suffered grade 3 or worse gastrointestinal hemorrhage. More bleeding events and anemia were found in patients with cervical cancer following SHR-1701 therapy [16], which might be attributed to different tumor types, prior treatments (such as radiotherapy), and/or complications. It has been reported that TGF-β signaling is involved in vascular development and stability [17, 18], but whether the occurrence of these bleeding events is caused by TGF-β inhibition and whether anemia is secondary to the bleeding events still need to be investigated.

Three deaths were judged possibly related to study treatment by the investigator. Two simultaneously suffered disease progression and associated complications, which might also relate to their deaths. The rest one had unexplained death after he dropped out of the study because of disease progression, so that conservatively judged as possibly related to study treatment.

The most favorable response with SHR-1701 was observed in the GC cohort with an ORR of 20.0% and DoR of 7.0 months. Two more patients had delayed response after iUPD, resulting in an ORR of 25.7% as determined by iRECIST. The OS rate at 12 months was as high as 54.5%. For patients treated with bintrafusp alfa, the ORR was 16%, DoR was 8.7 months, and the 12-month OS rate was 41% [19]. With regard to the approved 2nd- or 3rd-line therapies, including chemotherapy (taxanes or irinotecan), targeted therapy (apatinib or ramucirumab), and immune checkpoint inhibitor (PD-1/PD-L1 blockade) monotherapy, the ORR was about 20%, or even less with targeted therapy and immune checkpoint inhibitor monotherapy, with the median OS of 5 to 9 months [20–26]. Overall, SHR-1701 showed quite encouraging efficacy, which might provide a new choice for pretreated GC.

SHR-1701 also showed clinical activity in pretreated HNSCC, RCC, and UC cohorts, with an ORR of 20.0%, 20.0%, and 16.7%, which were comparable to immune checkpoint inhibitor monotherapy (13–17% [27–29], 25% [30], and 17–26% [31–34], respectively). Low or no response was seen in other cohorts. As some patients were still on treatment, the ORR might change with extended follow-up, and delayed response after iUPD might occur. For the above tumor types, further studies are warranted to screen the potential benefit population of SHR-1701 monotherapy or improve the efficacy by combination strategies.

It has been reported that bintrafusp alfa had a higher ORR of 30.5% in human papillomavirus (HPV)-related cancers, mainly including cervical cancer and HPV-positive HNSCC [35]. There were 10 HNSCC patients in this study. Most of these patients were diagnosed with nasopharyngeal carcinoma (7/10), for which HPV infection is not a predominant cause, and the HPV status in the rest three HNSCC patients was unknown. Whether HPV infection is associated with the response to SHR-1701 needs further investigations. Currently, a randomized, double-blind phase 3 study in patients with persistent, recurrent, or metastatic cervical cancer comparing SHR-1701 versus placebo in combination with platinum-based chemotherapy with or without bevacizumab biosimilar (NCT05179239) is underway.

We also aimed to identify biomarkers to determine patients who could benefit most from SHR-1701. Regardless of tumor types, patients with high PD-L1 CPS showed higher ORR, suggesting a certain predictive utility of PD-L1 expression. It had been reported that among gastric and gastroesophageal junction cancer patients with PD-L1 CPS ≥1, immune checkpoint inhibitor monotherapy provided a numerically higher ORR than those with PD-L1 CPS <1 (15.5% [23/148] vs 6.4% [7/109]) [20]. While dual inhibitor bintrafusp alfa showed similar ORR (17.4% [4/23] vs 16.7% [2/12]) [19]. In our study, SHR-1701 achieved a numerically higher ORR in patients with PD-L1 CPS ≥1 compared with <1 (21.7% vs 12.5%), and the rate was even higher in patients with CPS ≥5 (45.5%) and ≥10 (55.6%). It indicated that PD-L1 expression could partly predict the efficacy of SHR-1701 in GC. The value would be further confirmed in follow-up studies.

SMAD2 and SMAD3 are downstream transcription factors critical in the TGF-β pathway. Upon phosphorylation, SMAD2 and SMAD3 accumulate in the nucleus, form trimeric SMAD complexes with SMAD4, and further interact with varied cofactors to control downstream gene expression [36, 37]. We firstly found a correlation of pSMAD2 level in tumor cells at baseline with a trend towards better ORR (H-score ≥235 vs <235: 36.4% vs 6.3%). Our results suggested that SHR-1701 might inhibit the SMAD2-dependent TGF-β pathway that contributes to tumor progression and immunosuppressive microenvironment. Due to the exploratory nature and small number of patients, these preliminary findings must be interpreted cautiously but highlight further investigations.

The main limitations of this study are typical of early-phase clinical trials. Most clinical-expansion cohorts had a small sample size and required further investigations. The lack of a control arm makes it difficult to contextualize findings in the GC cohort relative to the historical comparator. In addition, the effects of SHR-1701 on TGF-β2 and TGF-β3 trapping need to be investigated. Based on the early sign demonstrated in this study, we have initiated a randomized, double-blind, placebo-controlled phase 3 study in GC patients assessing the addition of SHR-1701 on first-line chemotherapy (ClinicalTrials.gov, NCT04950322).

## Conclusions

Overall, SHR-1701 showed an acceptable safety profile and encouraging antitumor activity in advanced malignancies. Albeit early, the data showed promising efficacy signals of SHR-1701 in advanced or metastatic GC. The PD-L1 expression and tumor cell pSMAD2 level might contribute in better patient selection, which needs future validation.

## Supplementary Information

**Additional file 1: Figure S1.** Study design. **Figure S2.** Tumor response of patients in the dose-escalation and dose-expansion phase. **Figure S3.** Percentage change from baseline in target lesion tumour burden over time in patients with select tumors at the recommended dose (30 mg/kg q3w). **Figure S4.** Receiver operating characteristic curve analysis of pSmad2 level in tumor cells for ORR per RECIST v1.1. **Table S1.** Pharmacokinetic parameters following a single infusion. **Table S2.** Summary of treatment-related adverse events and tumor response by dose in the dose-escalation and dose-expansion phase. **Table S3.** Characteristics of patients in clinical expansion cohorts by tumor types. **Table S4.** Serious treatment-related adverse events. **Table S5.** Tumor response by PD-L1 expression in all clinical expansion cohorts and in gastric cancer cohort. **Table S6.** Associations between tumor response and pSMAD2 level in clinical expansion cohorts.

## Data Availability

Individual deidentified participant data that underlie the results reported in this article will be considered for sharing after the product and indication has been approved by major health authorities (e.g., China National Medical Products Administration, US Food and Drug Administration, European Medicines Agency, etc.). Data may be requested 24 months after study completion. Qualified researchers should submit a proposal to the corresponding authors outlining the reasons for requiring the data. The sponsor will provide individual deidentified participant data if the proposal is approved, provided that the requestor signs a data-access agreement. The use of data must comply with the requirements of the Human Genetics Resources Administration of China and other country or region-specific regulations.

## Abbreviations

AUC: Area under the curve
BTC: Biliary tract cancer
CBR: Clinical benefit rate
CPS: Combined positive score
CR: Complete response
DCR: Disease control rate
DoR: Duration of response
GC: Gastric cancer
HCC: Hepatocellular carcinoma
HNSCC: Head and neck squamous cell carcinoma
HPV: Human papillomavirus
H-score: Histochemical score
iRECIST: Modified RECIST 1.1 for immune-based therapeutics
iUPD: Immune unconfirmed progressive disease
ORR: Objective response rate
OS: Overall survival
PD-1: Programmed death receptor 1
PD-L1: Programmed death ligand 1
PFS: Progression-free survival
PR: Partial response
RCC: Renal cell carcinoma
RECIST: Response Evaluation Criteria in Solid Tumors
SCC: Squamous cell carcinoma
TGF-β: Transforming growth factor-β
TRAE: Treatment-related adverse event
UC: Urothelial carcinoma
