# Supplementary material for: Bifunctional anti-PD-L1/TGF-βRII agent SHR-1701 in advanced solid tumors: a dose-escalation, dose-expansion, and clinical-expansion phase 1 trial
Source: BMC Med. 2022 Oct 25;20:408. doi: 10.1186/s12916-022-02605-9 (PMC9594927; doi:10.1186/s12916-022-02605-9)
Supplement: Supplementary file 1 — Additional file 1: Figure S1. Study design. Figure S2. Tumor response of patients in the dose-escalation and dose-expansion phase. Figure S3. Percentage change from baseline in target lesion tumour burden over time in patients with select tumors at the recommended dose (30 mg/kg q3w). Figure S4. Receiver operating characteristic curve analysis of pSmad2 level in tumor cells for ORR per RECIST v1.1. Table S1. Pharmacokinetic parameters following a single infusion. Table S2. Summary of treatment-related adverse events and tumor response by dose in the dose-escalation and dose-expansion phase. Table S3. Characteristics of patients in clinical expansion cohorts by tumor types. Table S4. Serious treatment-related adverse events. Table S5. Tumor response by PD-L1 expression in all clinical expansion cohorts and in gastric cancer cohort. Table S6. Associations between tumor response and pSMAD2 level in clinical expansion cohorts. [file 12916_2022_2605_MOESM1_ESM.docx]

**Supplementary**

**Table of contents**

[Figure S1. Study design 2](#_Toc101354629)

[Figure S2. Tumor response of patients in the dose-escalation and dose-expansion phase. 3](#_Toc101354630)

[Figure S3. Percentage change from baseline in target lesion tumour burden over time in patients with select tumors at the recommended dose (30 mg/kg q3w) 5](#_Toc101354631)

[Figure S4. Receiver operating characteristic curve analysis of pSmad2 level in tumor cells for ORR per RECIST v1.1. 6](#_Toc101354632)

[Table S1. Pharmacokinetic parameters following a single infusion 7](#_Toc101354633)

[Table S2 Summary of treatment-related adverse events and tumor response by dose in the dose-escalation and dose-expansion phase 8](#_Toc101354634)

[Table S3. Characteristics of patients in clinical expansion cohorts by tumor types 10](#_Toc101354635)

[Table S4. Serious treatment-related adverse events 12](#_Toc101354636)

[Table S5 Tumor response by PD-L1 expression in all clinical expansion cohorts and in gastric cancer cohort 13](#_Toc101354637)

[Table S6. Associations between tumor response and pSMAD2 level in clinical expansion cohorts 14](#_Toc101354638)

# Figure S1. Study design


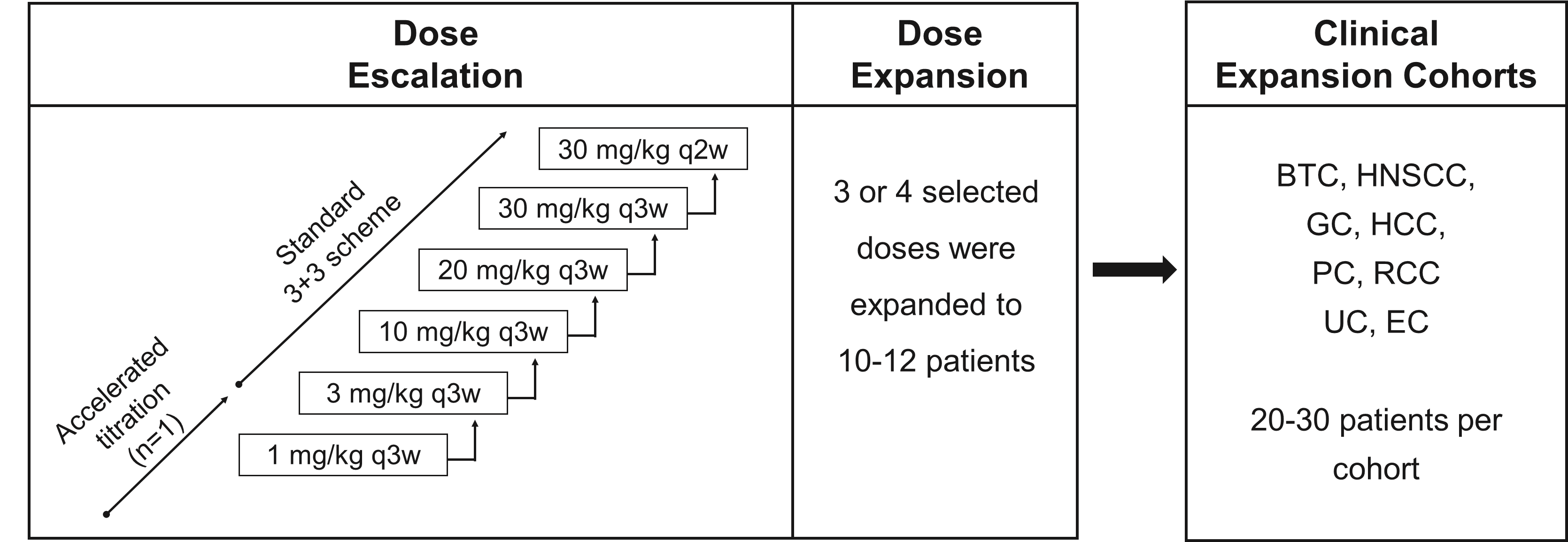


This was a multicenter, open-label, first-in-human phase I trial of SHR-1701 composed of a dose-escalation phase and dose-expansion phase in advanced solid tumors, followed by clinical expansion cohorts of selected tumor types (ClinicalTrials.gov, NCT03710265).

BTC, biliary tract cancer; HNSCC, head and neck squamous cell carcinoma; GC, gastric cancer; HCC, hepatocellular carcinoma; PC, pancreatic cancer; RCC, renal cell carcinoma; UC, urothelial carcinoma; EC, esophageal cancer.

# **Figure S2. Tumor response of patients in the dose-escalation and dose-expansion phase.**

**
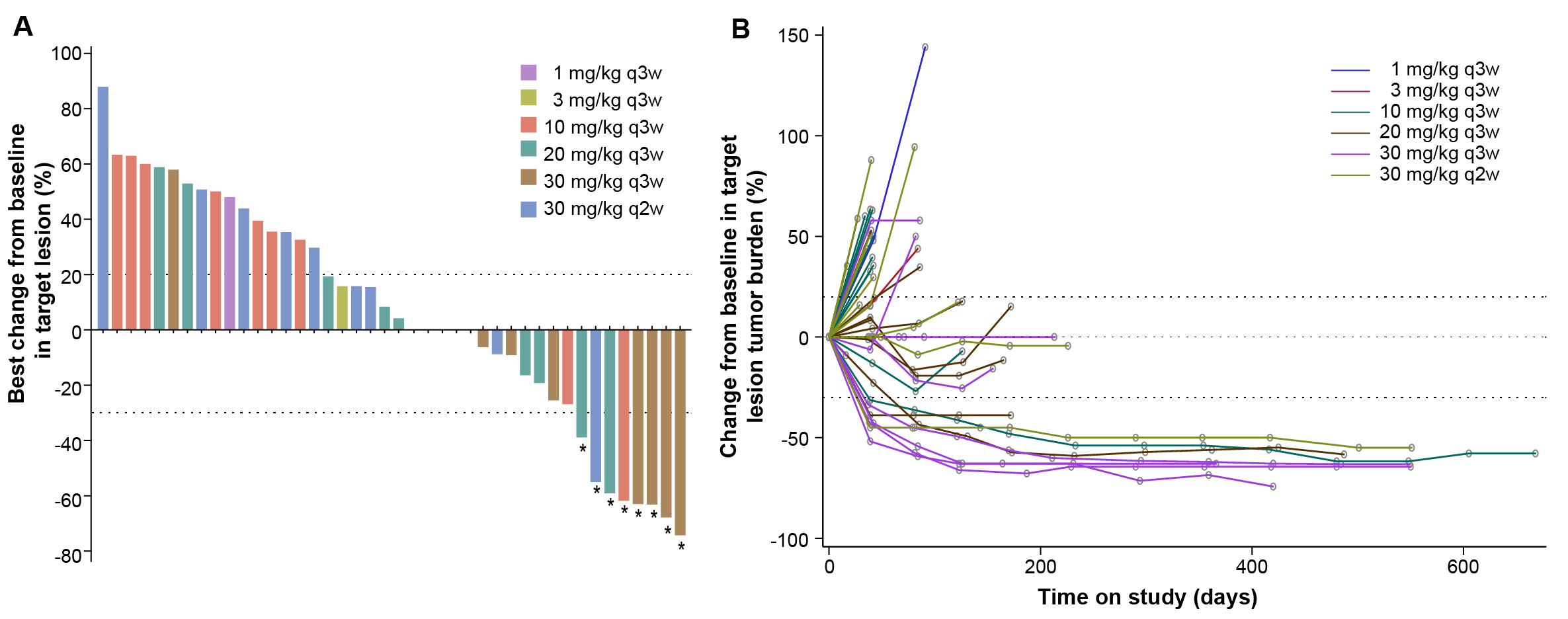
**

**A.** Waterfall plots of best overall response.

***** represent patients with partial reasponse, including 2 patients with non-small cell lung cancer, 1 pancreatic acinar cell carcinoma, 1 oesophageal squamous cell carcinoma, 1 hepatocellular carcinoma, 1 dMMR colorectal cancer, 1 renal caner, and 1 epiglottis cancer.

**B.** Percentage change from baseline in target lesion tumor burden over time.

**
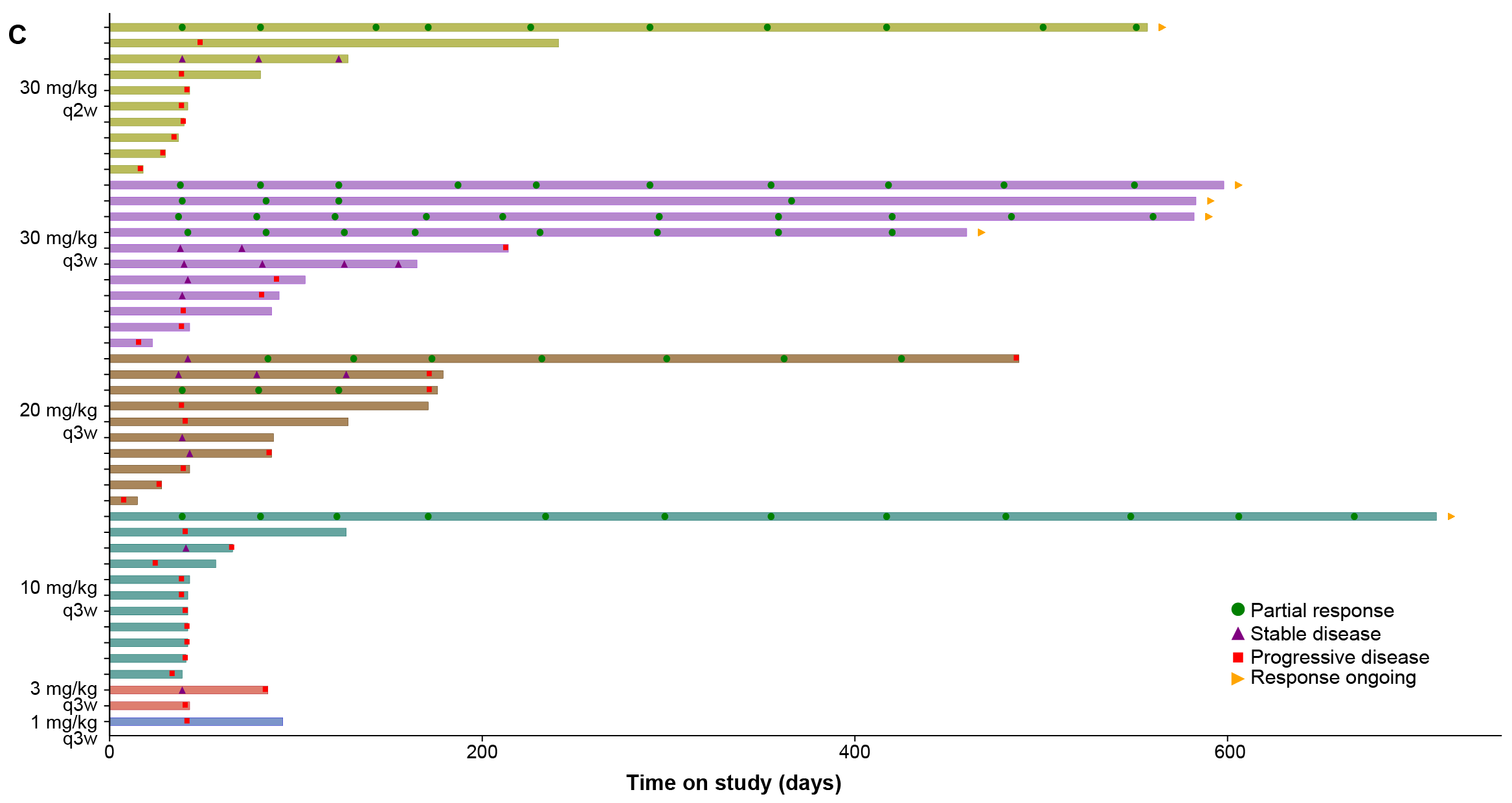
**

**C.** Tumor responses over time.

# **Figure S3.** Percentage change from baseline in target lesion tumour burden over time in patients **with select tumors at the recommended dose (30 mg/kg q3w)**


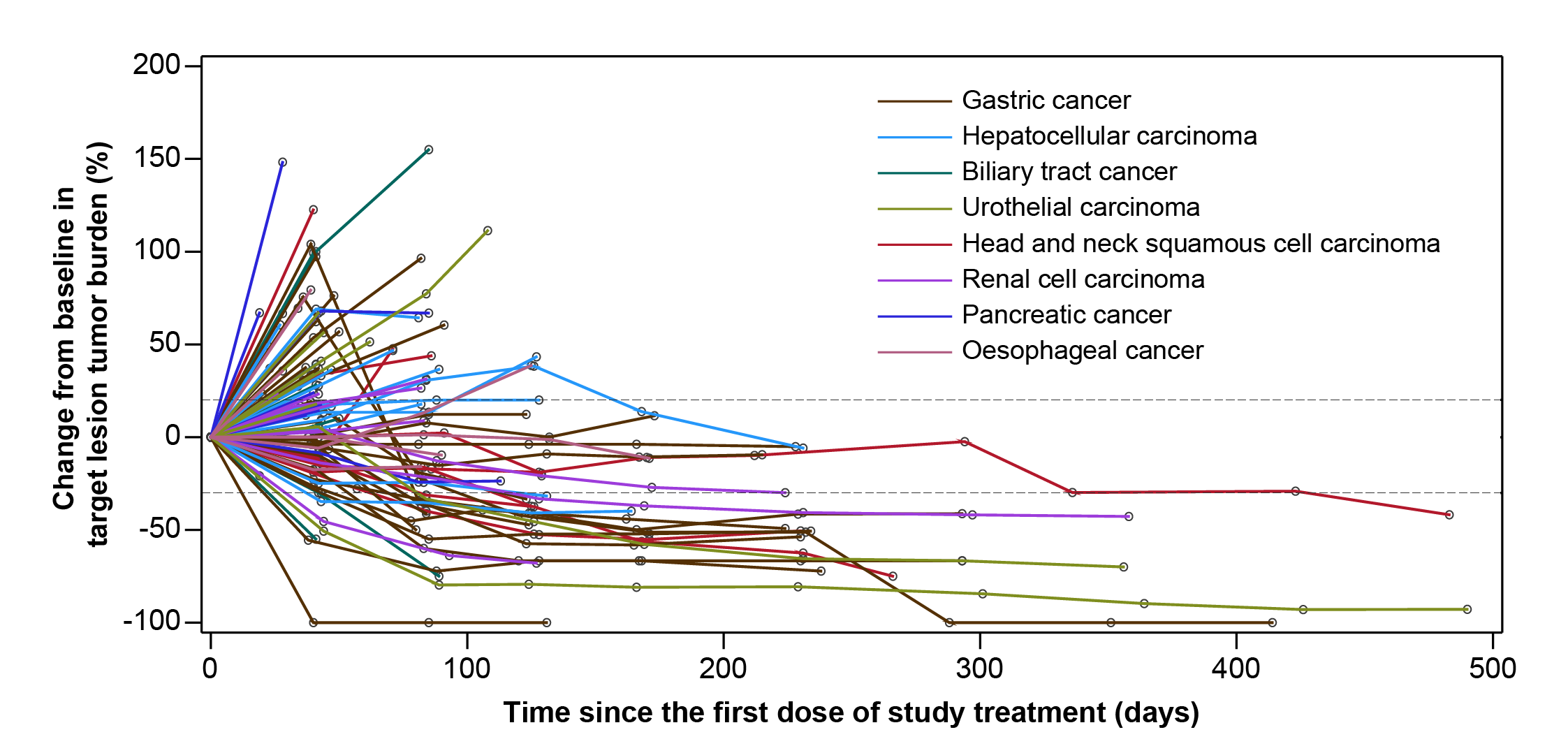


# **Figure S4. Receiver operating characteristic curve analysis of pSmad2 level** in tumor cells **for ORR per RECIST v1.1.**


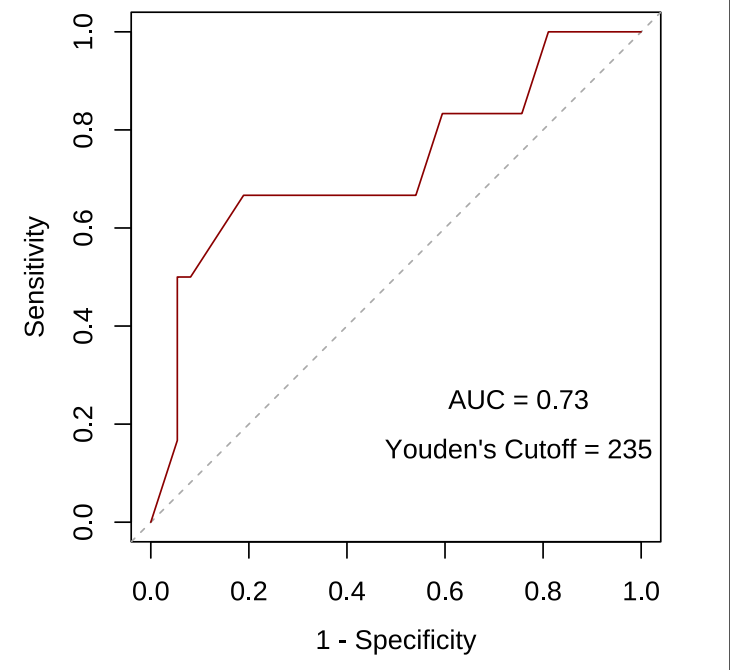


AUC, area under the curve; ORR, objective response rate.

# **Table S1. Pharmacokinetic** **parameters following a single infusion**

|  | **1 mg/kg q3w**  **(n=1)** | **3 mg/kg q3w**  **(n=3)** | **10 mg/kg q3w**  **(n=11)** | **20 mg/kg q3w**  **(n=12)** | **30 mg/kg q3w**  **(n=13)** | **30 mg/kg q2w**  **(n=10)** |
| --- | --- | --- | --- | --- | --- | --- |
| C_max_, geomean (%CVb), μg/mL | 24 | 90 (11.4) | 240 (19.3) | 409 (31.4) | 786 (37.1) | 846 (26.1) |
| T_max_, median (range), h | 2.00 | 2.97  (1.12-6.87) | 2.98  (1.18-48.53) | 1.68  (0.92-2.98) | 1.93  (0.75-24.07) | 1.83  (0.92-24.83) |
| AUC_inf_, geomean (%CVb), h·μg/mL | 3610 | 10200 (36.2) | 43100 (19.2) | 82500 (31.0) | 175000 (27.7) | 156000 (23.9) |
| AUC_last_, geomean (%CVb), h·μg/mL | 3330 | 9550 (30.6) | 39800 (18.4) | 59200 (60.8) | 110000 (65.4) | 110000 (24.2) |
| t_1/2_, geomean (%CVb), d | 6.0 | 4.6 (52.0) | 6.5 (17.7) | 7.2 (35.3) | 8.4 (16.7) | 6.3 (26.8) |
| CL, geomean (%CVb), mL/h | 15.8 | 17.0 (28.6) | 14.1 (20.1) | 14.3 (29.7) | 10.8 (26.0) | 12.0 (27.5) |
| Vss, geomean (%CVb), L | 3.25 | 2.70 (22.7) | 3.21 (15.7) | 3.67 (42.0) | 3.11 (22.3) | 2.61 (17.9) |

Geomean, geometric mean; CVb, coefficient of variation of Bowen ratio; C_max_, maximum serum concentration; T_max_, time to reach maximum serum concentration; AUC_inf_, area under the curve from time zero to the infinity; AUC_last_, AUC from time zero to the last concentration measurement; t_1/2_, half-life; CL, clearance; Vss, volume of distribution.

# **Table S2** **Summary of treatment-related adverse events and tumor response by dose in the dose-escalation and dose-expansion phase**

|  | **1 mg/kg q3w**  **(n=1)** | **3 mg/kg q3w**  **(n=3)** | **10 mg/kg q3w**  **(n=11)** | **20 mg/kg q3w**  **(n=12)** | **30 mg/kg q3w**  **(n=13)** | **30 mg/kg q2w**  **(n=10)** | **All patients**  **(N=50)** |
| --- | --- | --- | --- | --- | --- | --- | --- |
| **Safety**, n (%) | | | | | | | |
| Any grade | 1 (100.0) | 3 (100.0) | 8 (72.7) | 11 (91.7) | 10 (76.9) | 8 (80.0) | 41 (82.0) |
| Grade ≥3 | 0 | 0 | 1 (9.1) | 3 (25.0) | 4 (30.8) | 3 (30.0) | 11 (22.0) |
| Serious | 0 | 1 (33.3) | 2 (18.2) | 3 (25.0) | 1 (7.7) | 2 (20.0) | 9 (18.0) |
| Leading to treatment interruption | 0 | 1 (33.3) | 3 (27.3) | 1 (8.3) | 4 (30.8) | 3 (30.0) | 12 (24.0) |
| Leading to treatment discontinuation | 0 | 0 | 0 | 0 | 1 (7.7) | 1 (10.0) | 2 (4.0) |
| **Efficacy** | | | | | | | |
| BOR, n (%) | | | | | | | |
| Partial response | 0 | 0 | 1 (9.1) | 2 (16.7) | 4 (30.8) | 1 (10.0) | 8 (16.0) |
| Stable disease | 0 | 1 (33.3) | 1 (9.1) | 3 (25.0) | 4 (30.8) | 1 (10.0) | 10 (20.0) |
| Progressive disease | 1 (100) | 1 (33.3) | 9 (81.8) | 5 (41.7) | 3 (23.1) | 8 (80.0) | 27 (54.0) |
| Not evaluable | 0 | 1 (33.3) | 0 | 2 (16.7) | 2 (15.4) | 0 | 5 (10.0) |
| ORR, % (95% CI) | 0  (0-97.5) | 0  (0-70.8) | 9.1  (0.2-41.3) | 16.7  (2.1-48.4) | 30.8  (9.1-61.4) | 10.0  (0.3-44.5) | 16.0  (7.2-29.1) |
| DCR, % (95% CI) | 0  (0-97.5) | 33.3  (0.8-90.6) | 18.2  (2.3-51.8) | 41.7  (15.2-72.3) | 61.5  (31.6-86.1) | 20.0  (2.5-55.6) | 36.0  (22.9-50.8) |

BOR, best overall response; ORR, objective response rate; DCR, disease control rate.

# **Table S3. Characteristics of patients in clinical expansion cohorts by tumor types**

|  | **Clinical Expansion Cohorts** | | | | | | | |
| --- | --- | --- | --- | --- | --- | --- | --- | --- |
|  | **Gastric cancer**  **(n=35)** | **Hepatocellular carcinoma**  **(n=21)** | **Biliary tract cancer**  **(n=13)** | **Urothelial carcinoma**  **(n=12)** | **Head and neck squamous cell carcinoma**  **(n=10)** | **Renal cell carcinoma**  **(n=10)** | **Pancreatic cancer**  **(n=10)** | **Oesophageal cancer**  **(n=10)** |
| **Age, median (range), years** | 61 (31-74) | 51 (21-68) | 55 (31-73) | 55 (46-72) | 58 (39-69) | 57 (22-71) | 58 (42-69) | 64 (47-74) |
| **Sex, n (%)** | | | | | | | | |
| Male | 28 (80.0) | 19 (90.5) | 9 (69.2) | 11 (91.7) | 9 (90.0) | 8 (80.0) | 6 (60.0) | 8 (80.0) |
| Female | 7 (20.0) | 2 (9.5) | 4 (30.8) | 1 (8.3) | 1 (10.0) | 2 (20.0) | 4 (40.0) | 2 (20.0) |
| **ECOG performance status, n (%)** | | | | | | | | |
| 0 | 4 (11.4) | 7 (33.3) | 2 (15.4) | 2 (16.7) | 2 (20.0) | 2 (20.0) | 3 (30.0) | 1 (10.0) |
| 1 | 31 (88.6) | 14 (66.7) | 11 (84.6) | 10 (83.3) | 8 (80.0) | 8 (80.0) | 7 (70.0) | 9 (90.0) |
| **No. of organs of metastases, n (%)** | | | | | | | | |
| 0 | 0 | 2 (9.5) | 0 | 0 | 3 (30.0) | 0 | 0 | 1 (10.0) |
| 1 | 14 (40.0) | 8 (38.1) | 5 (38.5) | 3 (25.0) | 0 | 1 (10.0) | 3 (30.0) | 4 (40.0) |
| 2 | 13 (37.1) | 5 (23.8) | 4 (30.8) | 2 (16.7) | 2 (20.0) | 1 (10.0) | 4 (40.0) | 4 (40.0) |
| 3 | 5 (14.3) | 5 (23.8) | 2 (15.4) | 1 (8.3) | 5 (50.0) | 2 (20.0) | 0 | 1 (10.0) |
| 4 or more | 3 (8.6) | 1 (4.8) | 2 (15.4) | 6 (50.0) | 0 | 6 (60.0) | 3 (30.0) | 0 |
| **Lines of prior anticancer therapies, n (%)** | | | | | | | | |
| 0 | 0 | 0 | 0 | 1 (8.3) | 1 (10.0) | 1 (10.0) | 0 | 0 |
| 1 | 16 (45.7) | 17 (81.0) | 11 (84.6) | 10 (83.3) | 5 (50.0) | 7 (70.0) | 3 (30.0) | 9 (90.0) |
| 2 | 19 (54.3) | 4 (19.0) | 2 (15.4) | 1 (8.3) | 4 (40.0) | 2 (20.0) | 7 (70.0) | 1 (10.0) |
| **Prior therapy, n (%)** | | | | | | | | |
| Chemotherapy | 35 (100) | 6 (28.6) | 13 (100) | 11 (91.7) | 9 (90.0) | 1 (10.0) | 10 (100) | 10 (100) |
| Targeted therapy | 10 (28.6) | 17 (81.0) | 1 (7.7) | 1 (8.3) | 2 (20.0) | 8 (80.0) | 1 (10.0) | 2 (20.0) |
| Immunotherapy | 0 | 0 | 0 | 0 | 0 | 1 (10.0) | 0 | 0 |
| Others | 0 | 2 (9.5) | 0 | 1 (8.3) | 0 | 0 | 1 (10.0) | 1 (10.0) |

ECOG, Eastern Cooperative Oncology Group.

# **Table S4. Serious treatment-related adverse events**

|  | **All Patients (N=171)** | |
| --- | --- | --- |
|  | **Any Grade** | **Grade 3-5** |
| **Any** | 25 (14.6) | 13 (7.6) |
| Unknown death | 2 (1.2) | 2 (1.2) |
| Pneumonia | 2 (1.2) | 1 (0.6) |
| Vomiting | 2 (1.2) | 0 |
| Immune-mediated lung disease | 2 (1.2) | 0 |
| T1DM | 1 (0.6) | 1 (0.6) |
| Hyponatremia | 1 (0.6) | 1 (0.6) |
| Hypercalcemia | 1 (0.6) | 1 (0.6) |
| Asthenia | 1 (0.6) | 1 (0.6) |
| Blood creatinine increased | 1 (0.6) | 1 (0.6) |
| Lipase increased | 1 (0.6) | 1 (0.6) |
| Nausea | 1 (0.6) | 1 (0.6) |
| Gastrointestinal hemorrhage | 1 (0.6) | 1 (0.6) |
| Immune-mediated hepatic disorder | 1 (0.6) | 1 (0.6) |
| Anemia | 1 (0.6) | 1 (0.6) |
| Pyrexia | 1 (0.6) | 0 |
| Alanine aminotransferase increased | 1 (0.6) | 0 |
| Platelet count decreased | 1 (0.6) | 0 |
| Diarrhea | 1 (0.6) | 0 |
| Immune-mediated enterocolitis | 1 (0.6) | 0 |
| Hepatic function abnormal | 1 (0.6) | 0 |
| Pneumonitis | 1 (0.6) | 0 |
| Interstitial lung disease | 1 (0.6) | 0 |

# **Table S5 Tumor response by PD-L1 expression in all clinical expansion cohorts and in** gastric cancer **cohort**

|  | **All clinical expansion cohorts** | **Gastric cancer cohort** |
| --- | --- | --- |
| **PD-L1 CPS** | | |
| ≥1 *vs* <1 | 20.8% (15/72, 12.2-32.0) *vs* 7.5% (3/40, 1.6-20.4), *P*=0.105 | 21.7% (5/23, 7.5-43.7) *vs* 12.5% (1/8, 0.3-52.7), *P*=1.0 |
| ≥5 *vs* <5 | 34.1% (14/41, 20.1-50.6) *vs* 5.6% (4/71, 1.6-13.8), *P*<0.001 | 45.5% (5/11, 16.7-76.6) *vs* 5.0% (1/20, 0.1-24.9), *P*=0.013 |
| ≥10 *vs* <10 | 40.6% (13/32, 23.7-59.4) *vs* 6.3% (5/80, 2.1-14.0), *P*<0.001 | 55.6% (5/9, 21.2-86.3) *vs* 4.5% (1/22, 0.1-22.8), *P*<0.001 |

ORR are shown in % (n/N, 95% CI). Tumor responses were assessed per RECIST v1.1.

ORR, objective response rate; PD-L1, programmed death ligand 1; CPS, combined positive score; CI, confidence interval.

# **Table S6. Associations between tumor response** **and pSMAD2 level in clinical expansion cohorts**

| **pSMAD2 level in immune cells** | |
| --- | --- |
| AUC | 0.48 |
| H-score ≥65 *vs* <65 | 16.7% (3/18) *vs* 12.0% (3/25); *P*=0.683 |
| **pSMAD2 level** **in tumor cells** | |
| AUC | 0.73 |
| H-score ≥235 *vs* <235 | 36.4% (4/11) *vs* 6.3% (2/32); *P*=0.029 |

ORR are shown in % (n/N); *P*. Tumor responses were assessed per RECIST v1.1.

AUC, area under the curve; ORR, objective response rate; H-score, histochemical score.
